# VEGFR2 regulates endothelial differentiation of colon cancer cells

**DOI:** 10.1186/s12885-017-3578-9

**Published:** 2017-08-30

**Authors:** Zhiyong Liu, Lisha Qi, Yixian Li, Xiulan Zhao, Baocun Sun

**Affiliations:** 1 Department of Pathology, Tianjin Medical University Cancer Institute and Hospital, Tianjin, 300060 China; 2 Department of Pathology, Tianjin Medical University, Tianjin, 300070 China; 3 The Key Laboratory of Tianjin Cancer Prevention and Treatment, Tianjin, 300060 China; 4 National Clinical Research Center for Cancer, Tianjin, 300060 China

**Keywords:** VEGFR2, VE-cadherin, Vasculogenesis, Colon cancer

## Abstract

**Background:**

Recent studies suggested that cancer stem-like cells contribute to tumor vasculogenesis by differentiating into endothelial cells. However, such process is governed by still undefined mechanism.

**Methods:**

At varying differentiation levels, three representative colon cancer cells were cultured in endothelial-inducing conditioned medium: human colon cancer cells HCT116 (HCT116) (poorly differentiated), SW480 (moderately differentiated), and HT29 (well differentiated). We tested for expression of endothelial markers (cluster of differentiation (CD) 31, CD34, and vascular endothelial (VE)-cadherin and their ability to form tube-like structures in 3D culture. We also observed VEGF secretion and expressions of endothelial markers and VEGFRs in HCT116 cells under hypoxia to simulate physiological conditions. In in vitro and in xenotransplantation experiments, VE growth factor receptor 2 (VEGFR2) antagonist SKLB1002 was used to test effect of VEGFR2 in endothelial differentiation of HCT116 cells. Expression levels of VEGFR2 and VE-cadherin were assessed by immunohistochemistry of human colon cancer tissues to evaluate clinicopathological significance of VEGFR2.

**Results:**

After culturing in endothelial-inducing conditioned medium, poorly differentiated HCT116 cells expressed endothelial markers and formed tube-like structure in vitro. HCT116 cells secreted more endogenous VEGF and expressed higher VEGFR2 under hypoxia. SKLB1002 impaired endothelial differentiation in vitro and xenotransplantation experiments, suggesting a VEGFR2-dependent mechanism. Increased expression of VEGFR2 correlated with differentiation, metastasis/recurrence, and poor prognosis in 203 human colon cancer samples. Positive correlation was observed between VEGFR2 and VE-cadherin expression.

**Conclusions:**

VEGFR2 regulates endothelial differentiation of colon cancer cell and may be potential platform for anti-angiogenesis cancer therapy.

## Background

Angiogenesis is one of the hallmarks in tumor growth, expansion, and progression [1]. In surrounding, pre-existing vascular network, endothelial cells form new irregular blood vessels and supply tumors with nutrients and oxygen [2]. Although drugs were identified as crucial therapeutic strategy in multiple and different solid tumors, they are not very effective and may elicit more aggressive tumor phenotypes [3, 4]. Over the last few years, studies identified heterogeneous tumor vasculature mechanisms, including vessel co-option, recruitment of endothelial precursor cells, and vasculogenic mimicry (VM) [5–7]. Tumor cells play important roles in angiogenesis. Tumor cells secrete pro-angiogenic growth factors and cytokines to induce angiogenesis; they also have stem cell properties and transdifferentiate into cells with endothelial phenotypes and directly participate in tumor vasculogenesis. Stem-like cells in glioblastoma contribute proportion of endothelial cells by endothelial differentiation [8, 9]. Moreover, through VM, tumor cells form fluid conducting networks in melanoma, lung, breast, ovary, and prostate cancers [10–14]. Our previous study also demonstrated that VM exists in colon cancer, and its occurrence is strongly associated with undesirable clinical outcomes [15]. Both angiogenesis and vasculogenesis are involved in tumor vascularization. Thus, both processes should be considered in development of antitumor therapies aimed at tumor blood supply.

Many cellular factors and molecular events drive tumor stem-like cells to express endothelial phenotypes; these factor and events include vascular endothelial (VE)-cadherin, stromal cell-derived factor 1 (SDF-1), and Twist1-Bmi1 and epithelial-mesenchymal transition (EMT), hypoxia, or oxygen-glucose deprivation [16–21]. However, despite these numerous variables, we still need to investigate exact mechanism of expression of concerned cells to further define potential targets.

Several researchers highlighted roles of paracrine factors released by cancer cells in targeting receptors on endothelial cell surface [22, 23]. Tumor cells express high levels of VEGF-A, a major player of VEGF family, which binds and activates VE growth factor receptor (VEGFR) 1 and VEGFR2 with high affinity. VEGFR2 is expressed mostly in endothelial cells and is up-regulated in tumor vasculature. Shalaby showed that VEGFR2 gene knockdown mice died because of insufficient vasculogenesis [24]. Lyden et al. described that VEGFR2 is also expressed in hematopoietic stem cells, which are possible progenitors of endothelial cells [25]. VEGFR2 also interacts with “angiogenic switch,” VE-cadherin [26]. These results indicate that VEGFR2 is essential for differentiation of endothelial precursor cells and vasculogenesis. However, further studies still need to clarify the role of VEGFR2 in inducing endothelial-differentiation of tumor stem-like cells.

In this study, we cultured colon cancer cells in endothelial-inducing conditioned medium and observed that poorly differentiated human colon cancer cells (HCT116) could express endothelial markers (cluster of differentiation (CD) 31, CD34, and VE-cadherin) and had increased ability to form tube-like structures in 3D culture in vitro. To mimic tumor microenvironment, we further cultured HCT116 cells under hypoxia and noted that cells secreted more endogenous VEGF and expressed higher VEGFR2. Antagonizing VEGFR2 by SKLB1002 in HCT116 cells impaired endothelial differentiation, as shown by in vitro and xenotransplantation experiments. In addition, we studied clinicopathological and prognostic significance of VEGFR2 in 203 human colon cancer samples and its correlation with VE-cadherin.

## Methods

### Cell culture reagents and animals

The human colon cancer cell lines HT29 (No.3111C0001CCC000109), SW480 (No.3111C0001CCC000166), HCT116 (No.3111C0001CCC000331) were obtained from the Cell Resource Center at the Institute of Basic Medical Sciences, Chinese Academy of Medical Sciences/Peking Union Medical College (Beijing, China). Cells were cultured in Iscove Modified Dulbecco Medium (IMDM) with 10% FBS (Hyclone, Logan, Utah, USA) and with or without VEGF (10 ng/ml, R&D Systems Inc., Minneapolis, MN, USA), EGF (10 ng/ml, R&D Systems Inc.) and b-FGF(5 ng/mL, R&D Systems Inc.). SKLB1002 was obtained from Selleck Chemicals (Houston, TX, USA). For in vitro assays, SKLB1002 was prepared initially as a 20 mmol/L stock solution in DMSO. Stock solution was diluted in the relevant assay media, and 0.1% DMSO served as a vehicle control. In some in vitro experiments, HCT116 cells were treated with SKLB1002 at 10 ng/mL for 24 h. For in vivo study in mice, SKLB1002 was suspended in 35% (*v*/v) polyethylene glycol solution containing 5% (*v*/v) DMSO and dosed at 0.1 mL/10 g of body weight. Experiments under hypoxic conditions (1% O2) were performed in hypoxic workstation Invivo2 400 (Biotrace International) for 24 h in some in vitro experiments.

### In vitro three-dimensional (3-D) coculture

Tube-structure-forming ability was tested by using 3D culture in vitro. Matrigel (0.1 mL/well) was applied on the 24-well culture plate and incubated at 37 °C for half an hour. After been trypsinized and suspended in the complete medium at 2.5 × 105cells/mL, the cells were plated onto the surface of Matrigel at 1 mL/well, and incubated at 37 °C for 48 h. Ten fields were counted for the graphical representation of the tube formation assays.

### ELISA

Cells were cultured in serum-free conditions, and the culture supernatants were collected after 72 h. Levels of VEGF protein Secretion in the supernatants were measured by using a commercially availableVEGF-A Quantikine ELISA kit (R&D Systems). Each sample was assayed in triplicate.

### Immunofluorescence

Antibodies to CD34, CD31, and VE-cadherin were from Abcam (Cambridge, UK). Antibodies to goat anti-rabbit and goat anti-mouse IgG-FITC were from Santa Cruz Biotechnology (Santa Cruz, CA, USA). Alexa Fluor 488 and 546 were from Molecular Probes (Eugene, OR, USA). Briefly, HCT116 cells cultured on sterile glass cover slips were fixed with 4% paraformaldehyde, quenched with 50 mmol/L NH4Cl, permeabilized in 0.2% Triton X-100, and blocked in 3% BSA. The slips were incubated with the primary antibodies overnight at 4 °C, labeled with the specific secondary antibodies for 1 h in the dark, counterstained with DAPI, mounted, and viewed with fluorescent microscopy (Olympus).

### Immunoblotting

Cells were lysed and Protein extracts (50–100 μg/lane) was separated in a 10% SDS-polyacrylamide gel electrophoresis and transferred on polyvinylidene difluoride membranes. Blots were blocked and incubated with primary antibodies over night at 4 °C, probed with secondary antibody (Santa Cruz Biotechnology), and visualized with ECL Western blot substrate (Millipore). The following antibodies were used: anti–CD31, anti-CD34, anti VE-cadherin, anti–phosphorylated VEGFR2, anti-FAK, anti-ERK (cell signaling technology), anti-phosphorylated ERK1/2 (cell signaling technology), anti–phosphorylated FAK (cell signaling technology), anti-VEGFR2(Santa Cruz Biotechnology), anti-VEGFR2(Santa Cruz Biotechnology), anti-VEGFR3(Santa Cruz Biotechnology), and anti-β-actin (Santa Cruz Biotechnology). The western blots representative data are from 3 experiments.

### Xenograft mouse model

Twenty female athymic (nu/nu) nude mice (4–5 weeks old) were obtained from Wei Tong Li Hua Experimental Animal Company (Beijing, China). The mice were randomly and evenly divided into two groups and given 1 × 10^6^ HCT116 cells by subcutaneous injection in left groin. After 10 days of inoculation, mice bearing tumors around 50 mm3, the SKLB1002 treatments were started. The control group was injected with the same amount of vehicle solution. The dosing schedules were SKLB1002 100 mg/kg/d or vehicle once a day intraperitoneally. Tumor size was measured every 5 days for 30 days. Tumor volumes were calculated using the following formula: volume = (length × width^2^)/2. At the end of experiment, mice were euthanized. Tumor samples were formalin fixed, paraffin embedded, and processed for hematoxylin & eosin (H&E) and immunohistochemical analysis.

### Clinical samples

Tissue samples of colon cancer were harvested from 203 patients who had undergone surgery for colon cancer in Tianjin Medical University Cancer Institute and Hospital (Tianjin, China) between January 2002 and December 2004. None of the patients had received any chemotherapy or radiotherapy before their operation. Data of clinicopathological parameters were obtained from patients’ clinical records and pathological reports. The follow-up time ranged from 2 to 109 months with a median of 47.1 months.

### Hematoxylin & eosin staining and the evaluation of the non-necrosis area

H&E staining were performed to examine the necrosis of tumor mass of Xenograft mice. Tissue samples were sectioned (thickness, 5 μm) and deparaffinized in xylene. Tissue sections were stained with hematoxylin and eosin, cleared in xylene and mounted on slides using neutral balsam. The photos were captured by the software Image-Pro Plus (Media Cybernetics, MD, USA). Based on the different color of the necrotic and non-necrotic areas, the software calculated the level of non-necrosis (as a percentage of the total area relative to the entire histological section). The results were presented as mean and standard deviation.

### Immunohistochemical staining

Typically, the sections were pretreated with microwaves, blocked, and incubated with antibodies (VEGFR2,1:100 or VE-cadherin, 1:100) overnight at 4 °C, then were immunostained with HRP-conjugated antibody. Signals were revealed using 3,3-diaminobenzidine buffer as substrate. PBS was used as the negative control. The expression of VEGFR2 and VE-cadherin was analyzed only histologically in neoplastic epithelial cells. The staining intensity of sections was graded on a scale from 0 to 2 (0 for no staining, 1 for weak immunoreactivity, 2 for strong immunoreactivity). Percentage immunoreactivity was scored on a scale from 0 to 3 (0 for no positive cells, 1 for <25% of cells being positive, 2 for 25% to 50% of cells being positive, and 3 for >50% of cells being positive). The two scores were multipled to obtain a composite expression score. Either VEGFR2 or VE-cadherin expression was classified as negative (score = 0), weakly positive (score = 1, 2, or 3), or strongly positive (score = 4, 5, or 6). The sections were scored blindly by two observers using a microscope at ×200 magnification.

### Statistical analysis

SPSS v.16.0 software (SPSS Inc., Chicago, IL, USA) was used for data analysis. The associations between VEGFR2 and clinicopathologic parameters and the differential expression of VE-cadherin between different VEGFR2 expression level groups were assessed with Fisher’s exact test and chi-square test. Differences or correlations between groups were assessed by the Mann–Whitney U-test, Student’s t-test and Pearson’s correlation test. Survival analysis was carried out according to Kaplan–Meier. Differences in survival curves were assessed using the log rank test. Significance was set at *P* < 0.05.

## Results

### Endothelial-inducing conditioned medium induced endothelial differentiation of HCT116 cells

**Fig. 1:**
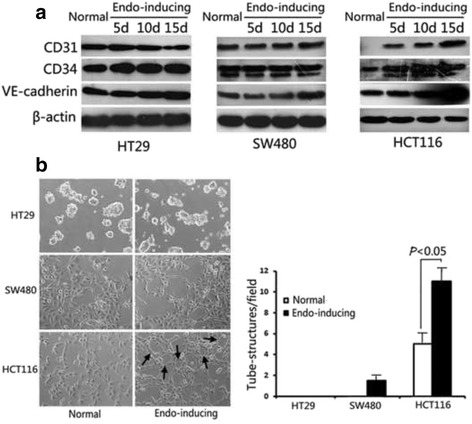
Endothelial-inducing conditioned medium induced expressions of CD31, CD34, and VE-cadherin and promoted HCT116 cells to form tube-like structures. **a** Protein expressions of CD31, CD34, and VE-cadherin in HT29, SW480 or HCT116 after cultured for 5, 10 or 15 days in endothelial-inducing conditioned medium were determined by western blotting analysis. Representative data of three experiments are shown. **b** The tube-like structures formed by HT29, SW480 or HCT116 cells in endothelial-inducing conditioned or normal 3D culture (left). Black arrows indicate the typical tube-like structures. Scale bar: 100 μm; Quantitative analysis of the mean number of tube-like structures and shown as mean ± SD (right)

To study whether colon adenocarcinoma cells can differentiate into endothelial cells at different levels of differentiation, we cultured three representative colon cancer cells, including HCT116 (poorly differentiated), SW480 (moderately differentiated), and HT29 (well differentiated), in endothelial-inducing conditioned medium (basal medium with 10 ng/mL EGF, 10 ng/mL VEGF, and 5 ng/mL) for 5, 10, and 15 days and examined expressions of endothelial markers, such as CD31, CD34, and VE-cadherin. As shown in Fig. 1a, endothelial-inducing conditioned medium did not significantly influence expressions of CD31, CD34, and VE-cadherin in both HT29 and SW480 cells. However, HCT116 cells acquired increased expression of endothelial cell markers when cultured for 15 days in the endothelial inducing medium, suggesting that HCT116 cells tend to differentiate toward endothelial lineage spontaneously during long-term culture.

Formation of capillary-like structures in matrigel is commonly used functional test for endothelial cells. When grown on matrigel, HCT116 cells cultured in endothelial-inducing conditioned medium developed capillary-like structures, whereas HT29 or SW480 cells failed to perform the same action (Fig. 1b). Collectively, these results suggest that poorly differentiated HCT116 cells possess endothelial-directional differentiation ability under endothelial-inducing microenvironment.

### VEGF secretion and VEGFR2 expression were elevated in HCT116 cells under hypoxia

**Fig. 2:**
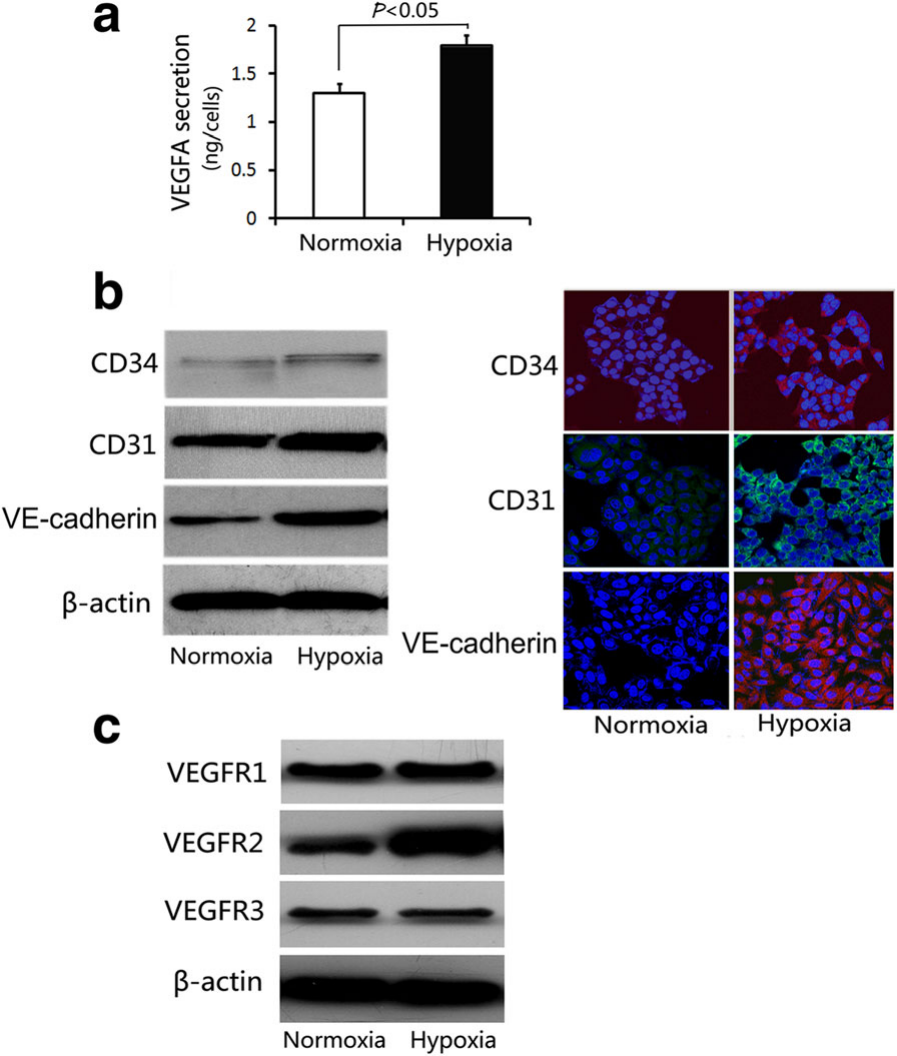
VEGF secretion and VEGFR2 expressions were elevated in HCT116 cells under hypoxia. **a** VEGFA secretion of HCT116 cells under hypoxia or normoxia were determined by ELISA analysis. The data are shown as mean ± SD, *n* = 3 in triplicate experiments. **b** Protein expressions of CD31, CD34, and VE-cadherin in HCT116 cells under hypoxia or normoxia were determined by western blotting (left) and immunofluorescent staining analysis (right). Representative data of three experiments are shown. **c** Protein expressions of VEGFR1, VEGFR2, and VEGFR3 in HCT116 cells under hypoxia or normoxia were determined by western blotting. Representative data of three experiments are shown

Hypoxia exists in microenvironment of majority of solid human tumors. This condition influences angiogenesis and vasculogenesis, which are notable aspects of tumor biology. To manipulate physiological circumstances, we cultured HCT116 cells under hypoxia (1% O2) in absence of growth factors or serum for 24 h. We discovered that cells secreted more VEGFA (Fig. 2a) and displayed increased expression of endothelial markers, such as CD31, CD34, and VE-cadherin (Fig. 2b).

VEGF signals are transduced through three different tyrosine kinase receptors, including VEGFR1, VEGFR2, and VEGFR3. Among these receptors, VEGFR2 is expressed on endothelial cells, bone marrow-derived endothelial progenitor cells, and numerous tumor cell types. VEGFR2 was also reported as principal mediator of vascular development and maturation. We detected expression of VEGFRs and noted that VEGFR2 expression increased in HCT116 cells under hypoxia (Fig. 2c), suggesting that VEGFR2 possibly participates in endothelial differentiation of colon cancer cells.

### SKLB1002 inhibited tube-like structure formation in vitro and inhibited expression of VE-cadherin of HCT116 cells

**Fig. 3:**
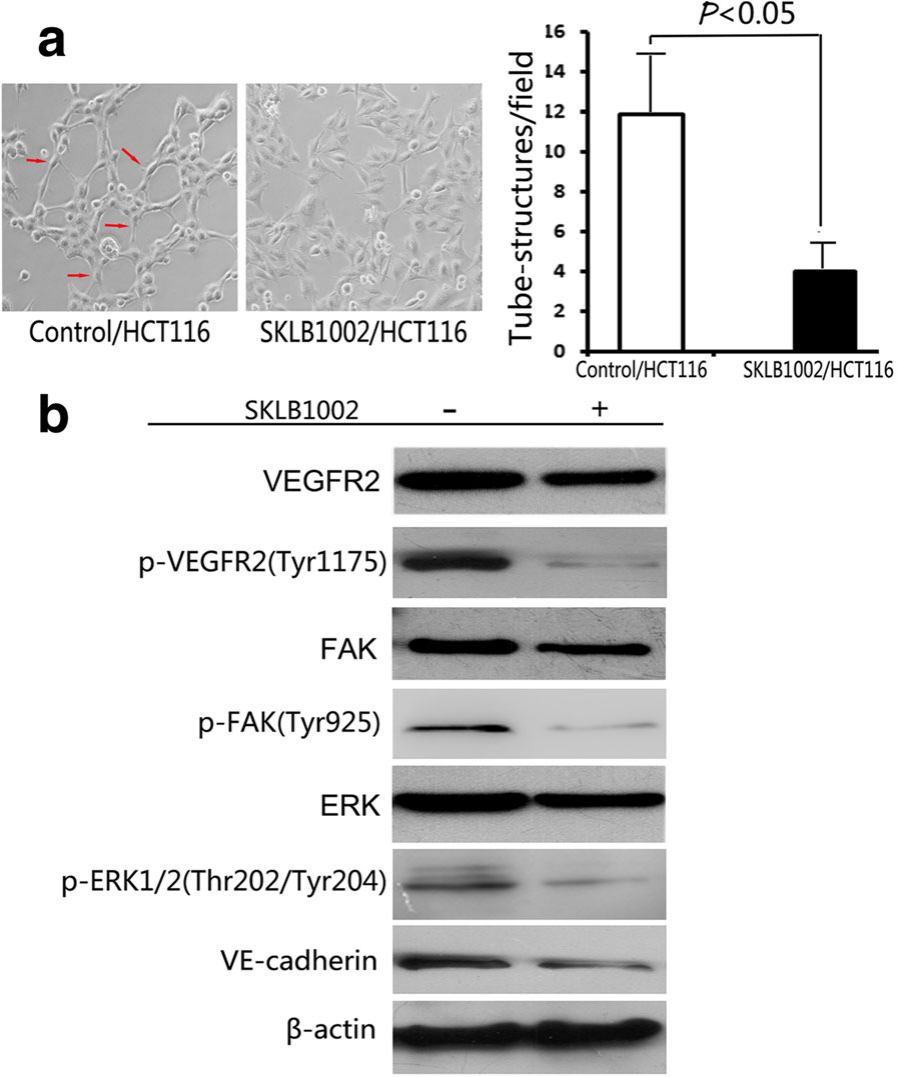
SKLB1002 inhibited tube-like structures formation and VE-cadherin expression of HCT116 cells. **a** The tube-like structures formed by HCT116 cells with or without SKLB1002 treatment (left). Red arrows indicate the typical tube-like structures. Scale bar: 100 μm; Quantitative analysis of the mean number of tube-like structures and shown as mean ± SD (right). **b** Protein expressions of VEGFR2, p-VEGFR2, FAK, p-FAK, ERK, p-ERK and VE-cadherin in HCT116 cells with or without SKLB1002 treatment were determined by western blotting analysis. Representative data of three experiments are shown

We also investigated the role of VEGFR2 in endothelial differentiation of HCT116 cells under hypoxia. SKLB1002 is ATP-competitive inhibitor of VEGFR2. This inhibitor can potentially inhibit VEGFR2 tyrosine kinase activity but cause few toxic effects in hosts. As shown in Fig. 3a, in presence of SKLB1002, differentiating HCT116 cells did not acquire endothelial ability to organize into tubular-like structures.

VEGF/VEGFR2 induces activation of downstream signaling components, including extracellular signal-regulated kinase (ERK) and focal adhesion kinase (FAK), which reportedly contributes to tumor angiogenesis. SKLB1002 significantly suppressed phosphorylation of VEGFR2, ERK, and FAK, suggesting that SKLB1002 exerted its function by directly targeting VEGFR2 and further antagonizing VEGFR2-mediated downstream signaling cascade. Moreover, SKLB1002 inhibited VE-cadherin expression (Fig. 3b), indicating the essential role of VEGFR2 in inducing endothelial differentiation of colon cancer cells.

### SKLB1002 suppressed in vivo tumor growth and VE-cadherin expression in animal models

**Fig. 4:**
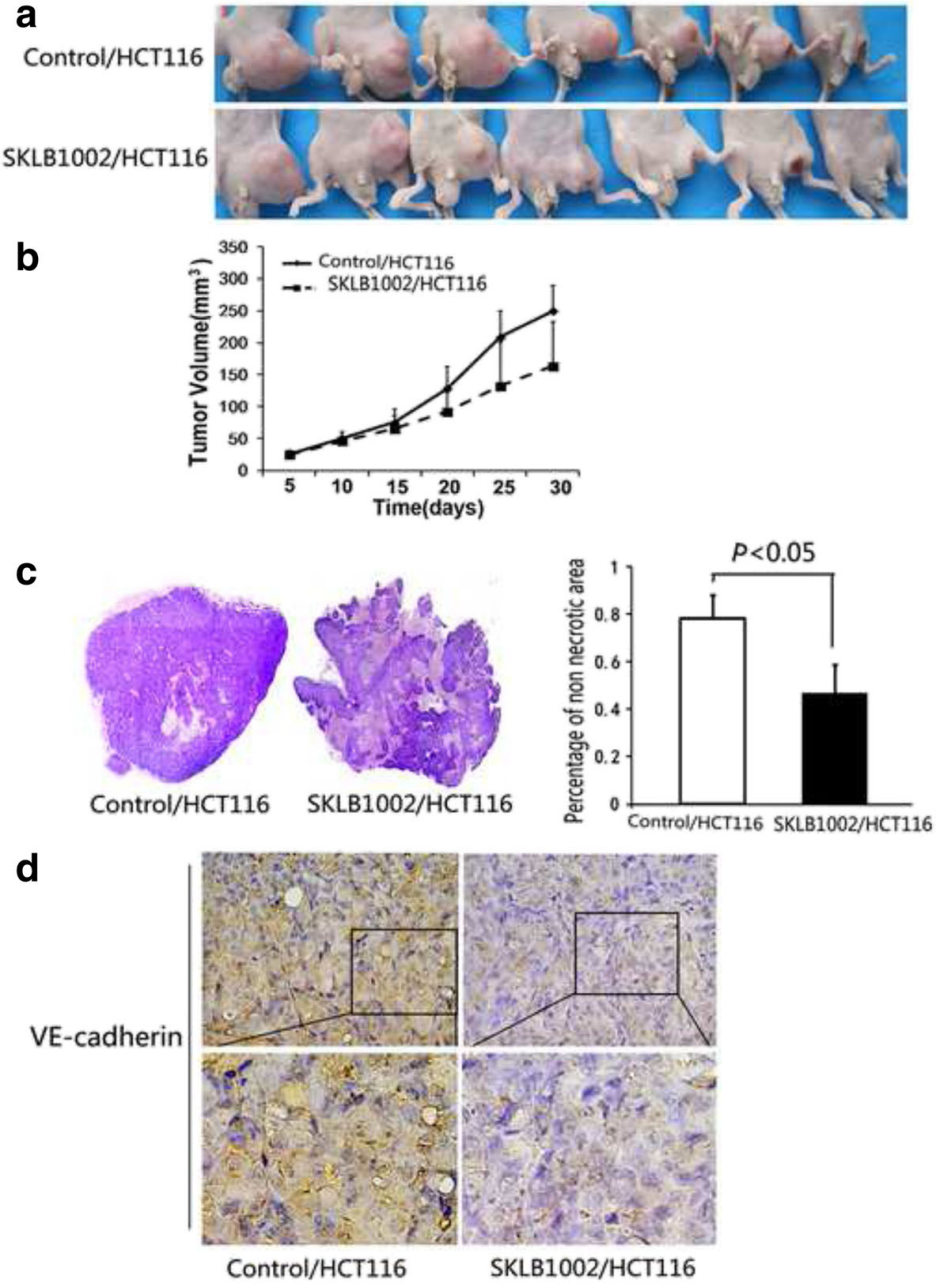
SKLB1002 inhibited in vivo tumor growth in HCT116 xenograft mouse model and decreased VE-cadherin expression in tumor tissues. **a** Photographs illustrating mice tumors derived from HCT116 cells with or without SKLB1002 treatment 30 days after inoculation. **b** The volume of the mice tumors derived from HCT116 cells was evaluated at 5-day intervals for 30 days. Data are shown as mean ± SD. **c** Hematoxylin & eosin staining photographs illustrating necrosis in mice tumor tissues derived from HCT116 cells with or without SKLB1002 treatment 30 days after inoculation (left). (×40). Quantitative analysis of the proportions of non-necrotic tissue in the largest section of tumor masses is shown as mean ± SD (right). **d** Expressions of VE-cadherin in mice tumor tissues derived from HCT116 cells with or without SKLB1002 treatment 30 days after inoculation were determined by immunohistochemical staining. (×400)

In malignancy, vascular supply is prerequisite for tumors growth, maintenance, and progression over time. To further confirm that SKLB1002 inhibited endothelial abilities of HCT116 cells, we used tumor xenograft models in nude mice. Pharmacological inhibition of VEGFR2 (SKLB1002) resulted in dramatic tumor shrinkage (Fig. 4a and b, *P*(day5^th^) = 0.436, *P*(day10^th^) = 0.406, *P*(day15^th^) = 0.266, *P*(day20^th^) = 0.071, *P*(day25^th^) = 0.050, *P*(day30^th^) = 0.045) and extensive necrosis (Fig. 4c). Consistent with in vitro results, immunohistochemical analyses showed remarkable decreased VE-cadherin expression in tumor sections from SKLB1002-stimulated cells compared with those of control group (Fig. 4d) (χ2 = 6.878, *P* = 0.032). In SKLB1002 treatment group, 8 mice showed negative VE-cadherin expression, 2 showed weakly VE-cadherin positive expression, no mice showed VE-cadherin strongly expression. Whereas in the mice without SKLB1002, 2 mice showed negative VE-cadherin expression, 7 showed weakly VE-cadherin positive expression, 1 mice showed VE-cadherin strongly expression.

### VEGFR2 expression increased in colon adenocarcinomas and correlated with clinical outcome of patients

**Fig. 5:**
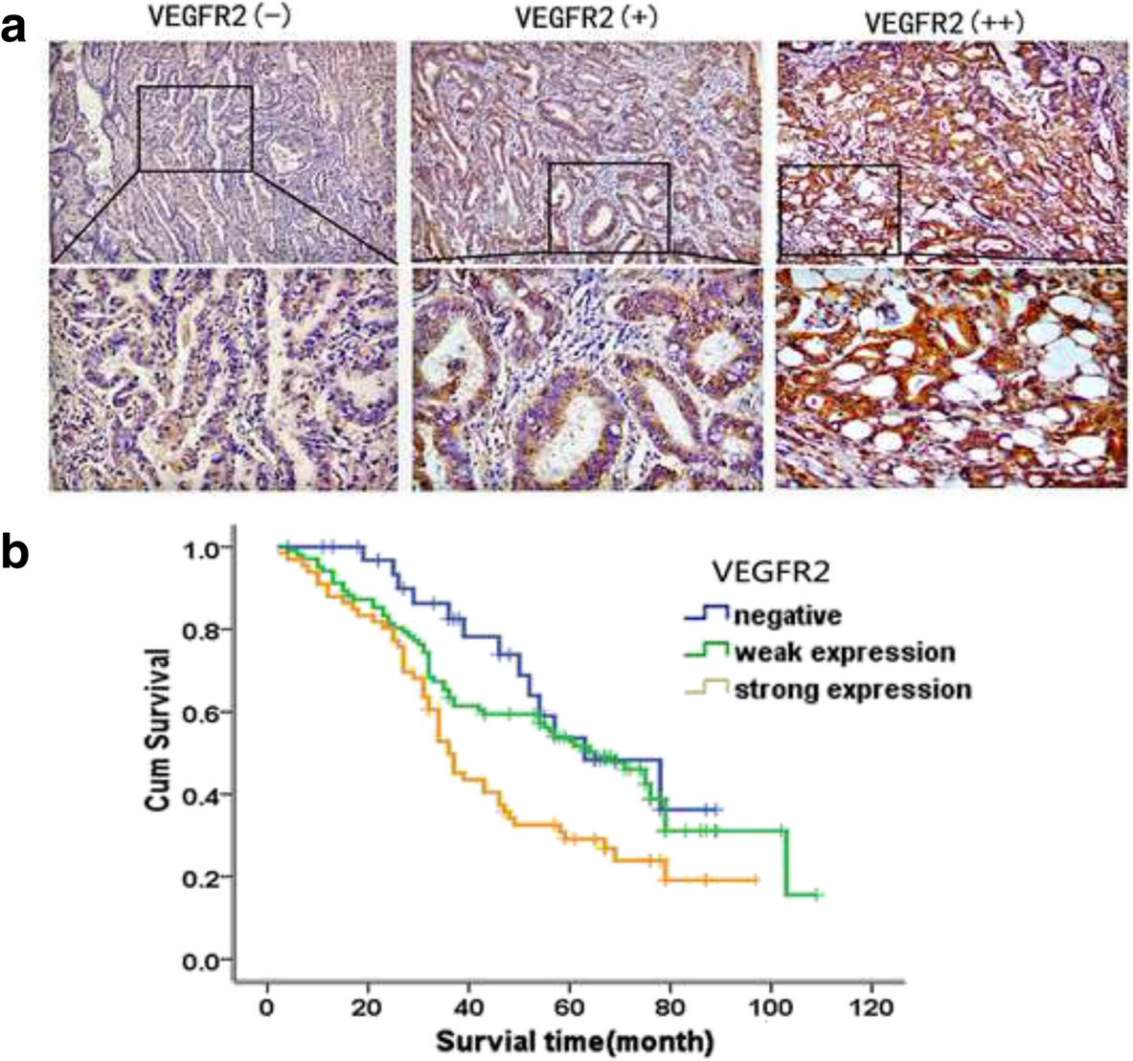
Expressions of VEGFR2 in colon cancer samles and Kaplan–Meier survival analysis of colon cancer patients. **a** Representative immunohistochemical staining photographs of human colon cancer samples with VEGFR2 negative (−), weak(+), and strong (++) expression. (× 200). **b** Kaplan–Meier survival analysis illustrating overall survival status in VEGFR2 negative (−), weak(+), and strong (++) groups of colon cancer patients

Expression patterns of VEGFR2 were examined on an array of 203 human colon adenocarcinoma cases. Among 203 samples, 168 (82.8%) showed positive VEGFR2 expression. Tumors were categorized as strong (++), weak (+), or negative (−) for VEGFR2 expression (Fig. 5a). Table 1 summarizes relationships between VEGFR2 levels and each clinicopathological parameter. In colon adenocarcinoma, VEGFR2 expression level increased with decreasing differentiation grade and metastasis. Positive VEGFR2 expression was observed in 10 of 14 (71.4%) well-differentiated patient samples, in 82 of 101 (81.2%) moderately differentiated patient samples, and 76 of 88 (86.4%) poorly differentiated patient samples. A total of 74 (36.5%) patients experienced metastasis. Patients with strong VEGFR2 expression had higher rate of metastasis (35/66, 53.0%) than those with weak (26/102, 25.5%) or negative VEGFR2 expression (13/35, 18.2%). Correlations were not observed between VEGFR2 expression level and patient age, gender, tumor size, or clinical stage.

**Table 1:** Correlation between VEGFR2 and clinicopathologic characteristics of patients with colon cancer

Viariant	Total	VEGFR2 expression			χ^2^	*P* value
		Negative (%)	Weak expression (%)	Strong expression (%)		
Age						
< 45	27	7(25.9)	12(44.4)	8 (29.6)	1.651	0.438
≥ 45	176	28 (15.9)	70(51.1)	58 (33.0)		
Sex						
Male	91	12(13.2)	46(50.5)	33 (36.3)	2.290	0.318
Female	112	23 (20.5)	56(50.0)	33(29.5)		
Tumor size						
≥ 5 cm	67	15 (22.4)	31(46.3)	21(31.3)	1.893	0.388
< 5 cm	136	20(14.7)	71(52.2)	45(33.1)		
Histological differentiation						
Well differentiated	14	4 (28.6)	8(57.1)	2 (14.3)	32.110	<0.001*
Moderately differentiated	101	19(18.8)	65(64.4)	17(16.8)		
Poorly differantiated	88	12(13.6)	29(33.0)	47 (53.4)		
Clinical stage						
TNMI	7	1(14.3)	4(57.1)	2(28.6)	7.518	0.276
TNMII	127	21(17.3)	71(55.9)	34(26.8)		
TNMIII	58	11(19.0)	23(39.7)	24(41.4)		
TNMIV	11	1 (9.1)	4(36.4)	6(54.5)		
Metastasis						
Absent	129	22(17.1)	76(58.9)	31 (24.0)	13.129	0.001*
Present	74	13(17.6)	26(35.1)	35 (47.3)		

*Significantly different

Kaplan–Meier survival analysis showed that total survival time for patients in VEGFR2 negative group was significantly longer than those in VEGFR2-weak expression or VEGFR2-strong expression groups (*P* = 0.007). VEGFR2 negative patients had average survival time of 64.3 months, whereas VEGFR2-weak expression and VEGFR2-strong expression groups had 62.8 and 47.0 months, respectively (Fig. 5b).

### Expression of VEGFR2 is concomitant with VE-cadherin expression

**Fig. 6:**
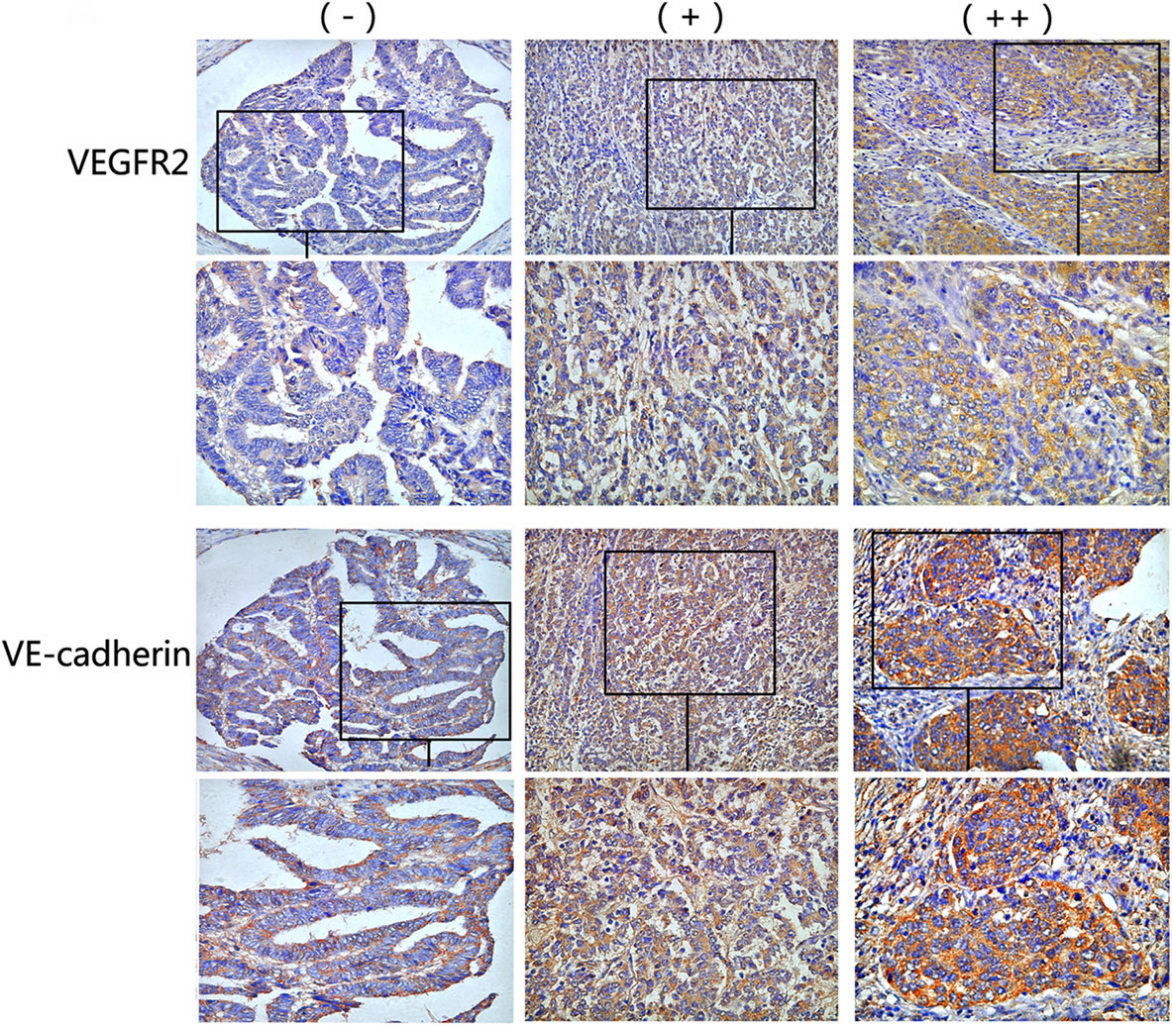
Expression of VEGFR2 is concomitant with VE-cadherin expression. Representative immunohistochemical VE-cadherin staining photographs of human colon cancer samples in VEGFR2 negative (−), weak(+), and strong (++) groups. (×200)

VEGFR2 and VE-cadherin expressions in 203 specimens were analyzed to assess relationship between VEGFR2 and endothelial differentiation of colon adenocarcinoma cells. VE-cadherin expression was higher (*P* < 0.05) in samples with weakly and strongly positive VEGFR2 expression than those with negative VEGFR2 expression (Table 2 and Fig. 6). These findings reinforced that VEGFR2 may be involved in endothelial differentiation of colon adenocarcinoma.

**Table 2:** Correlation between expression of VEGFR2 and VE-cadherin

Variant	Total	VEGFR2 expression			χ^2^	*P* value
		Negative (%)	Weak expression (%)	Strong expression (%)		
VE-cadherin expression					12.457	0.014*
Negative	55	16 (29.1)	28(50.9)	11 (20.0)		
Weak expression	59	11(18.6)	29(49.2)	19(32.2)		
Strong epression	89	8(9.0)	45(50.6)	36(40.4)		

*Significantly different

## Discussion

Colon cancer is one of the most common cancers worldwide. Although colon cancer diagnosis and treatment significantly advanced over the past two decades, five-year survival rate remains below 50%. Angiogenesis is important regulator of local and metastatic growth of colorectal cancer [27]. In specific subgroups of patients, survival benefit was attributed to endothelial-cell-specific chemotherapy by addition of anti-VEGF monoclonal antibody [28]. However, several reports indicated that this treatment may elicit more aggressive tumor phenotypes [3, 4]. Thus, anti-angiogenesis treatment should be developed based on deeper understanding of related pathogenesis and biological features.

In this study, after culturing in endothelial-inducing medium, HCT116 differentiated into cells with endothelial markers, including CD31, CD34, and VE-cadherin, and exhibited endothelial tube-like morphology. We placed HCT116 cells under hypoxia and in absence of growth factors or serum to manipulate physiological circumstances leading to development of low-oxygen conditions, which are caused by imbalance between supply and consumption. Under hypoxic stress, HCT116 cells showed increased VEGFA secretion and expression of endothelial cell-specific cell surface proteins. This result is consistent with reports of Wang et al. and Ricci-Vitiani et al., who described that subpopulation of endothelial cells within glioblastomas harbor the same somatic mutations identified within tumor cells [8, 9]. Interestingly, HT29 and SW480 did not show remarkable increased expression of endothelial markers and failed to successfully form tube-like structure compared with poorly differentiated HCT116 cells. We previously reported that most VM existed in poorly differentiated tissues in human colon cancer samples [15]. We propose that colon cancer cells can acquire typical endothelial cell characteristics, and this ability may be related to stem cell-like origins. Novel antiangiogenesis therapies should simultaneously target cancer cells with endothelial transdifferentiation capacity and endothelial cells.

VEGFs displayed different affinities for three receptor tyrosine kinases, including VEGFR1–3 [29]. Both stromal and cancer cells secrete VEGF-A, which binds VEGFR-2 to regulate multiple aspects of angiogenesis; such as aspects include endothelial cell development, survival, migration, and lumenization [30]. Although initially thought to be exclusively expressed by endothelial cells, VEGFR-2 is also expressed by different cancers [31–33]. Thus, VEGF/VEGFR-2 drives cancer neovascularization via both paracrine and autocrine effects. Our present data showed that under hypoxia stimulation, HCT116 cells expressed higher VEGFR2 but not VEGFR1 and VEGFR3. During embryonic development, mesodermal stem cells and angioblasts express VEGFR2, and they can differentiate into endothelial cells to form primitive vascular plexus-like structures. Our study supports a model wherein tumor cells mirror differentiations, which are observed in angioblasts, during physiological embryogenesis to contribute to neovascularization.

As endothelial cell–cell adhesion molecule, VE-cadherin is necessary for vascular integrity and regulates endothelial cell assembly into tubular structures [34]. Deletion or cytosolic truncation of VE-cadherin impairs remodeling and maturation of vascular networks [35, 36]. During endothelial transdifferentiation of cancer cells, VE-cadherin is identified as “endothelial switch” because tumor cells lacking VE-cadherin cannot form tube-like structure. In addition, VE-cadherin is implicated in tumorigenesis, suggesting potential of VE-cadherin as target in cancer treatment [37, 38]. Although previous studies did not indicate roles for VEGFR2 in regulating VE-cadherin expression, transmembrane domain of VEGFR2 is associated with VE-cadherin, which in turn is linked to β-catenin/α-catenin complex and activates Akt signaling [26, 39]. Blocking VEGFR2 with SKLB1002 inhibited VE-cadherin expression and prevented tube-structure formation of HCT116 cells in vitro. Studied nude mice also showed similar significant inhibitory effects of SKLB1002 on VE-cadherin expression. Remarkably, VEGFR2 expression significantly correlated with VE-cadherin expression on same tumor cells in human colon cancer samples, indicating that VEGFR2 possibly aids colon cancer cells in acquiring differentiation potential for endothelial cells.

VEGFR2 was largely and strongly expressed in cancer cells in 203 colon cancer tissues, suggesting that it may have another role in cancer cell biology aside from being a vasculature-restricted receptor. In addition, in accordance with a previous study, which demonstrated significant association of VEGFR2 expression with poor tumor histological differentiation in 128 colorectal adenocarcinoma tissues, the present study showed that VEGFR2 expression correlated significantly with differentiation, metastasis/recurrence, and poor diagnosis [40]. In this regard, with its capacity to enhance transdifferentiation of tumor cells into endothelial cells, VEGFR2 may be advantageous for tumor progression.

In summary, colon cancer cells can transdifferentiate along endothelial lineages, both morphologically and functionally. More importantly, this study indicated critical role of VEGFR2 in promoting endothelial differentiation of colon cancer cells. Results pose several implications for underlying importance of VEGFR2 as useful therapy target.

## Conclusions

Poorly differentiated HCT116 colon cancer cell in endothelial cell inducing conditions (specific media or hypoxia) results in endothelial phenotype. VEGFR2 regulates endothelial differentiation of colon cancer cell and may be potential platform for anti-angiogenesis cancer therapy.

## Abbreviations

CD31: Cluster of differentiation 31
CD34: Cluster of differentiation 34
EMT: Epithelial-mesenchymal transition
ERK: Extracellular signal-regulated kinase
FAK: Focal adhesion kinase
SDF-1: Stromal cell-derived factor 1
VE-cadherin: Vascular endothelial-cadherin
VEGF: Vascular endothelial growth factor
VEGFR: Vascular endothelial growth factor receptor
VM: Vasculogenic mimicry

## References

[1] Baish JW, Jain RK (1998). Cancer, angiogenesis and fractals. Nat Med.

[2] Carmeliet P, Jain RK (2000). Angiogenesis in cancer and other diseases. Nature.

[3] Soda Y, Myskiw C, Rommel A, Verma IM (2013). Mechanisms of neovascularization and resistance to anti-angiogenic therapies in glioblastoma multiforme. J Mol Med (Berlin, Germany).

[4] Xu Y, Li Q, Li XY, Yang QY, Xu WW, Liu GL (2012). Short-term anti-vascular endothelial growth factor treatment elicits vasculogenic mimicry formation of tumors to accelerate metastasis. J Exp Clin Cancer Res.

[5] Pezzella F, Gatter K (2015). Non-angiogenic tumours unveil a new chapter in cancer biology. J Pathol.

[6] Moschetta M, Mishima Y, Sahin I, Manier S, Glavey S, Vacca A, Roccaro AM, Ghobrial IM (1846). Role of endothelial progenitor cells in cancer progression. Biochim Biophys Acta.

[7] Qian CN (2013). Hijacking the vasculature in ccrcc--co-option, remodelling and angiogenesis. Nat Rev Urol.

[8] Ricci-Vitiani L, Pallini R, Biffoni M, Todaro M, Invernici G, Cenci T, Maira G, Parati EA, Stassi G, Larocca LM (2010). Tumour vascularization via endothelial differentiation of glioblastoma stem-like cells. Nature.

[9] Wang R, Chadalavada K, Wilshire J, Kowalik U, Hovinga KE, Geber A, Fligelman B, Leversha M, Brennan C, Tabar V (2010). Glioblastoma stem-like cells give rise to tumour endothelium. Nature.

[10] Hendrix MJ, Seftor EA, Seftor RE, Chao JT, Chien DS, Chu YW (2016). Tumor cell vascular mimicry: Novel targeting opportunity in melanoma. Pharmacol Ther.

[11] Williamson SC, Metcalf RL, Trapani F, Mohan S, Antonello J, Abbott B, Leong HS, Chester CP, Simms N, Polanski R (2016). Vasculogenic mimicry in small cell lung cancer. Nat Commun.

[12] Cui YF, Liu AH, An DZ, Sun RB, Shi Y, Shi YX, Shi M, Zhang Q, Wang LL, Feng Q (2015). Claudin-4 is required for vasculogenic mimicry formation in human breast cancer cells. Oncotarget.

[13] Su M, Xu X, Wei W, Gao S, Wang X, Chen C, Zhang Y (2016). Involvement of human chorionic gonadotropin in regulating vasculogenic mimicry and hypoxia-inducible factor-1alpha expression in ovarian cancer cells. Cancer Cell Int.

[14] Wang H, Lin H, Pan J, Mo C, Zhang F, Huang B, Wang Z, Chen X, Zhuang J, Wang D (2016). Vasculogenic mimicry in prostate cancer: The roles of epha2 and pi3k. J Cancer.

[15] Liu Z, Sun B, Qi L, Li H, Gao J, Leng X (2012). Zinc finger e-box binding homeobox 1 promotes vasculogenic mimicry in colorectal cancer through induction of epithelial-to-mesenchymal transition. Cancer Sci.

[16] Zhao N, Sun H, Sun B, Zhu D, Zhao X, Wang Y, Gu Q, Dong X, Liu F, Zhang Y (2016). Mir-27a-3p suppresses tumor metastasis and vm by down-regulating ve-cadherin expression and inhibiting emt: An essential role for twist-1 in hcc. Sci Rep.

[17] Kamprom W, Kheolamai P, Yaowalak U, Supokawej A, Wattanapanitch M, Laowtammathron C, Issaragrisil S (2016). Effects of mesenchymal stem cell-derived cytokines on the functional properties of endothelial progenitor cells. Eur J Cell Biol.

[18] Kirschmann DA, Seftor EA, Hardy KM, Seftor RE, Hendrix MJ (2012). Molecular pathways: Vasculogenic mimicry in tumor cells: Diagnostic and therapeutic implications. Clin Cancer Res.

[19] Yang D, Wang J, Xiao M, Zhou T, Shi X (2016). Role of mir-155 in controlling hif-1alpha level and promoting endothelial cell maturation. Sci Rep.

[20] Nakagomi T, Kubo S, Nakano-Doi A, Sakuma R, Lu S, Narita A, Kawahara M, Taguchi A, Matsuyama T (1962). Brain vascular pericytes following ischemia have multipotential stem cell activity to differentiate into neural and vascular lineage cells. Stem Cells.

[21] Chen HF, Huang CH, Liu CJ, Hung JJ, Hsu CC, Teng SC, Wu KJ (2014). Twist1 induces endothelial differentiation of tumour cells through the jagged1-klf4 axis. Nat Commun.

[22] Yun EJ, Lorizio W, Seedorf G, Abman SH, Vu TH (2016). Vegf and endothelium-derived retinoic acid regulate lung vascular and alveolar development. *American journal of physiology*. Lung Cell Mol Physiol.

[23] Lee YC, Gajdosik MS, Josic D, Clifton JG, Logothetis C, Yu-Lee LY, Gallick GE, Maity SN, Lin SH (2015). Secretome analysis of an osteogenic prostate tumor identifies complex signaling networks mediating cross-talk of cancer and stromal cells within the tumor microenvironment. Mol Cell Proteomics.

[24] Shalaby F, Rossant J, Yamaguchi TP, Gertsenstein M, Wu XF, Breitman ML, Schuh AC (1995). Failure of blood-island formation and vasculogenesis in flk-1-deficient mice. Nature.

[25] Lyden D, Hattori K, Dias S, Costa C, Blaikie P, Butros L, Chadburn A, Heissig B, Marks W, Witte L (2001). Impaired recruitment of bone-marrow-derived endothelial and hematopoietic precursor cells blocks tumor angiogenesis and growth. Nat Med.

[26] Coon BG, Baeyens N, Han J, Budatha M, Ross TD, Fang JS, Yun S, Thomas JL, Schwartz MA (2015). Intramembrane binding of ve-cadherin to vegfr2 and vegfr3 assembles the endothelial mechanosensory complex. J Cell Biol.

[27] Liu Z, Sun B, Qi L, Li Y, Zhao X, Zhang D, Zhang Y (2015). Dickkopf-1 expression is down-regulated during the colorectal adenoma-carcinoma sequence and correlates with reduced microvessel density and vegf expression. Histopathology.

[28] Tournigand C, Chibaudel B, Samson B, Scheithauer W, Vernerey D, Mesange P, Lledo G, Viret F, Ramee JF, Tubiana-Mathieu N (2015). Bevacizumab with or without erlotinib as maintenance therapy in patients with metastatic colorectal cancer (gercor dream; optimox3): A randomised, open-label, phase 3 trial. Lancet Oncol.

[29] Shibuya M (2002). Vascular endothelial growth factor receptor family genes: When did the three genes phylogenetically segregate?. Biol Chem.

[30] Baeriswyl V, Christofori G (2009). The angiogenic switch in carcinogenesis. Semin Cancer Biol.

[31] Lee TH, Seng S, Sekine M, Hinton C, Fu Y, Avraham HK, Avraham S (2007). Vascular endothelial growth factor mediates intracrine survival in human breast carcinoma cells through internally expressed vegfr1/flt1. PLoS Med.

[32] Silva SR, Bowen KA, Rychahou PG, Jackson LN, Weiss HL, Lee EY, Townsend CM, Evers BM (2011). Vegfr-2 expression in carcinoid cancer cells and its role in tumor growth and metastasis. *International journal of cancer*. J Int du cancer.

[33] Lichtenberger BM, Tan PK, Niederleithner H, Ferrara N, Petzelbauer P, Sibilia M (2010). Autocrine vegf signaling synergizes with egfr in tumor cells to promote epithelial cancer development. Cell.

[34] Vittet D, Buchou T, Schweitzer A, Dejana E, Huber P (1997). Targeted null-mutation in the vascular endothelial-cadherin gene impairs the organization of vascular-like structures in embryoid bodies. Proc Natl Acad Sci U S A.

[35] Luo Y, Radice GL (2005). N-cadherin acts upstream of ve-cadherin in controlling vascular morphogenesis. J Cell Biol.

[36] Giannotta M, Trani M, Dejana E (2013). Ve-cadherin and endothelial adherens junctions: Active guardians of vascular integrity. Dev Cell.

[37] Labelle M, Schnittler HJ, Aust DE, Friedrich K, Baretton G, Vestweber D, Breier G (2008). Vascular endothelial cadherin promotes breast cancer progression via transforming growth factor beta signaling. Cancer Res.

[38] Muramatsu F, Kidoya H, Naito H, Sakimoto S, Takakura N (2013). Microrna-125b inhibits tube formation of blood vessels through translational suppression of ve-cadherin. Oncogene.

[39] Yamaoka-Tojo M, Tojo T, Kim HW, Hilenski L, Patrushev NA, Zhang L, Fukai T, Ushio-Fukai M (2006). Iqgap1 mediates ve-cadherin-based cell-cell contacts and vegf signaling at adherence junctions linked to angiogenesis. Arterioscler Thromb Vasc Biol.

[40] Giatromanolaki A, Sivridis E, Koukourakis MI (2006). Angiogenesis in colorectal cancer: Prognostic and therapeutic implications. Am J Clin Oncol.

